# The Role of Virtual Consulting in Developing Environmentally Sustainable Health Care: Systematic Literature Review

**DOI:** 10.2196/44823

**Published:** 2023-05-03

**Authors:** Martha Pickard Strange, Amy Booth, Melissa Akiki, Sietse Wieringa, Sara E Shaw

**Affiliations:** 1 Nuffield Department of Primary Care Health Sciences University of Oxford Oxford United Kingdom; 2 University of Oslo Oslo Norway

**Keywords:** virtual consulting, environmental sustainability, systematic review, carbon footprinting, net zero, mobile phone

## Abstract

**Background:**

Health systems globally need to rapidly set and achieve targets for reaching net zero carbon emissions. Virtual consulting (including video- and telephone-based consulting) is regarded as one means by which this might be achieved, largely through reduced patient travel. Little is currently known about the ways in which forms of virtual consulting might contribute to the net zero agenda or how countries may develop and implement programs at scale that can support increased environmental sustainability.

**Objective:**

In this paper, we asked, *What is the impact of virtual consulting on environmental sustainability in health care?* and *What can we learn from current evaluations that can inform future reductions in carbon emissions?*

**Methods:**

We conducted a systematic review of published literature according to PRISMA (Preferred Reporting Item for Systematic Reviews and Meta-Analyses) guidelines. We searched the MEDLINE, PubMed, and Scopus databases using key terms relating to “carbon footprint,” “environmental impact,” “telemedicine,” and “remote consulting,” using citation tracking to identify additional articles. The articles were screened, and full texts that met the inclusion criteria were obtained. Data on the approach to carbon footprinting reported reductions in emissions, and the opportunities and challenges associated with the environmental sustainability of virtual consultations were extracted into a spreadsheet, analyzed thematically, and theorized using the Planning and Evaluating Remote Consultation Services framework to consider the various interacting influences, including environmental sustainability, that shape the adoption of virtual consulting services.

**Results:**

A total of 1672 papers were identified. After removing duplicates and screening for eligibility, 23 papers that focused on a range of virtual consulting equipment and platforms across different clinical conditions and services were included. The focus on the environmental sustainability potential of virtual consulting was unanimously reported through carbon savings achieved by a reduction in travel related to face-to-face appointments. The shortlisted papers used a range of methods and assumptions to determine carbon savings, reporting these using different units and across varied sample sizes. This limited the potential for comparison. Despite methodological inconsistencies, all papers concluded that virtual consulting significantly reduced carbon emissions. However, there was limited consideration of wider factors (eg, patient suitability, clinical indication, and organizational infrastructure) influencing the adoption, use, and spread of virtual consultations and the carbon footprint of the entire clinical pathway in which the virtual consultation was provided (eg, risk of missed diagnoses from virtual consultations that result in the need for subsequent in-person consultations or admissions).

**Conclusions:**

There is overwhelming evidence that virtual consulting can reduce health care carbon emissions, largely through reducing travel related to in-person appointments. However, the current evidence fails to look at system factors associated with implementing virtual health care delivery and wider research into carbon emissions across the entire clinical pathway.

## Introduction

### Background

The risks of climate change are now widely accepted. Current projections suggest that we could see increases of 3 to 4°C in global temperatures by 2100, with catastrophic effects [1,2]. Extreme weather events including heat waves and flooding confirm the urgency of the challenge. Given the relationship between health and the environment, many of the effects are being felt in people’s health, both directly (eg, heat-related deaths and rises in vector-borne infections [3]) and indirectly (eg, disrupted food systems [4], reduced access to health care facilities owing to extreme weather events, and impacts on transport systems [5]). Urgent action needs to be taken to reduce carbon emissions and radically reduce the impact on health and well-being [1,6-10].

Health systems are major contributors to climate change because of the large quantities of greenhouse gases (GHGs) they emit (eg, owing to patterns of energy use, waste disposal, and complex supply chains [11-16]). If the global health sector were a country, some estimates put it as the fifth-largest source of GHG emissions on the planet [8]. In the United Kingdom, the National Health Service (NHS) is responsible for approximately 4% of all carbon emissions and approximately a fifth of public sector emissions [8]. This not only pollutes the environment but also leads to increased rates of illness (eg, cardiovascular disease and asthma [17-20]), with knock-on effects on costs [21]. Challenges are compounded by the rise in acute and chronic diseases, increased patient load, declining workforce, and system fragmentation [22].

This concern with environmental sustainability is part of a wide-ranging agenda to address climate change; progress has been made in the last decade, including via intergovernmental agreements and actions aimed at reducing global emissions (eg, the Paris Agreement [2]). More urgently needs to be done [23].

In the United Kingdom, the Climate Change Act of 2008 introduced public and private sector obligations to meet carbon reduction targets [24,25]. In health care, this led to the NHS Net Zero strategy [8], with targets set across the United Kingdom to achieve net zero health services by 2045 (or earlier where possible) and strategic roadmaps and actions for local organizations. To achieve this, multiple solutions are needed to reconfigure the delivery of care and the wider supply chain [26]. Technology offers a potential route for change. This includes the use of virtual consulting, which involves synchronous use of telephone or video platforms when consulting, either between clinicians and patients or across facilities (eg, from primary to secondary care [27]). Previously a novel service development [28,29], the shock of the COVID-19 pandemic enabled rapid and large-scale changes to the way services were delivered [30-32], with telephone and video technology allowing clinicians and patients to connect remotely and ensure physical distancing and infection control. There is a well-established evidence base supporting telephone consulting [33]. More recent research has demonstrated high levels of feasibility and acceptability of virtual consulting, with reductions in travel contributing to reduced emissions [11,34-36].

A recent systematic review of the evidence on the carbon footprint of telemedicine [35] showed that telephone and video consulting offer potential benefits in terms of reducing emissions and that these are largely attributable to patient and staff travel. The review was framed in terms of low-carbon alternatives to standard care and showed significant reductions (between 0.70 and 372 kg of carbon dioxide equivalent [CO_2_e] per consultation) associated with reduced transport-associated emissions. This was a significant step forward in this small (but rapidly growing) area of research. However, the review paid limited attention to the adoption, routinization, and spread of video consulting (eg, [28,29,32,36]) and the ways in which wider organizational and system incentives and initiatives (eg, specific policies, operational resources, and pathway redesign) shape potential for environmental gains. Papers in the review tended to focus uncritically on patient travel, with little consideration of other potential mitigating factors limiting, or indeed contributing to, carbon emissions. Hence, although the published evidence to date has highlighted that a shift to virtual consulting could generate a positive impact on NHS-related carbon emissions, the net effect of such changes has not yet been established.

### Objectives

More intensive action on the environmental impact is required to enable health systems to meet long-term carbon footprint goals. Important learning remains to be gained on the role of remote consulting in meeting these goals. This paper therefore asked, *What is the impact of virtual consulting on environmental sustainability in health care?* and *What can we learn from current evaluations that can inform future reductions in carbon emissions?*

## Methods

This review forms part of a wider study on Remote by Default Primary Care [37] and builds on a program of work on virtual consulting undertaken before and during the COVID-19 pandemic [28,31,32,34,36]. The PRISMA (Preferred Reporting Item for Systematic Reviews and Meta-Analyses) guidelines [38] were consulted throughout to guide the review. PRISMA-P (Preferred Reporting Items for Systematic Review and Meta-Analysis Protocols) was used to draft the protocol for this review (unpublished).

### Eligibility Criteria

Inclusion and exclusion criteria are summarized in Textbox 1.

**Summary of inclusion and exclusion criteria.**

**Inclusion criteria**

- Peer-reviewed, full-text papers presenting original research
- Papers reporting telephone and synchronous video consulting services (using video-based conferencing via website- or app-based formats)
- Papers published from 2009 (this being the earliest date of publication identified in a previous systematic review)

**Exclusion criteria**

- Papers where environmental impact was not assessed
- Papers reporting telephone and synchronous video consulting services established or routinized in mainstream health care settings
- Papers reporting exclusively pilot or partial video consulting services
- Papers reporting web-based e-consulting services
- Papers not reported in English

### Information Sources and Search Strategy

Our search strategy was developed with the help of a research librarian and used a mix of keywords and Medical Subject Headings—Major Headings. We searched MEDLINE, PubMed, and Scopus in November 2021 and updated the search again in August 2022. Search terms were identified from relevant published literature using 2 categories of keywords covering environmental sustainability (eg, “carbon footprint” and “environmental impact”) and virtual consulting (eg, “telemedicine” and “remote consultation”). Filters were applied to limit the results to published peer-reviewed articles and the English language only. A full list of the search terms can be found in Multimedia Appendix 1.

Using the same search terms and eligibility criteria, as outlined in the paragraph above and in Textbox 1, we identified additional studies via other methods. One author (AB) maintains an EndNote library of studies on environmental issues in health care, which was searched for any additional papers relating to telemedicine. This database was put together through historical searches on environmental impact and sustainability in health care using proximity searching (within 5 words) to bring search terms on environmental impact and sustainability closer together. We used citation tracking to identify further papers focusing on virtual consulting and sustainable health care. Finally, we included 1 organizational document reporting research on the environmental impact of telemedicine, identified via Google Scholar.

### Selection Process

The search results were imported into EndNote and duplicates were removed. Each record was initially independently and manually reviewed by title and abstract by 2 authors (MPS and MA). Where there was doubt about whether articles met the inclusion criteria based on the title and abstract, the full-text version was obtained and screened by 3 authors (AB, MPS, and MA), and inclusion or exclusion was verified by the project lead (SES). At each stage, articles were eliminated if they did not meet the inclusion criteria. In 1 case [39] where it was unclear whether the paper met the inclusion criteria given that it was published as a report, the paper was discussed, consensus was reached that it did meet the criteria, and it was included.

### Data Collection Process and Synthesis

At a descriptive level, we first extracted data on study location or setting, study timing, clinical focus, definition of telehealth, service model and type of technology, research design, and key findings. Second, as the majority of papers focused on carbon footprinting, we extracted specific data on the approach to carbon footprinting, methodology used, and any reductions in carbon emissions achieved in the service or project. We elected not to convert the carbon footprint data to the same units or to extrapolate the carbon savings per individual consultation in each study but to present and work with the measurements presented in each paper. Each paper worked with different units, different types and setup of virtual consultations, different health care services, and facilities that were located at different distances from patients, and we felt that converting the studies to the same units would be misleading and detract from our analysis of the various methods that the authors used to determine carbon footprints in their papers. Finally, we extracted data on how each paper conceptualized environmental sustainability and the opportunities and challenges perceived to be provided by virtual consulting services. This process was undertaken independently by 2 authors (MA and MPS) before being checked by SES. All data were extracted into a Microsoft Excel spreadsheet and are presented in tables.

**Figure 1:**
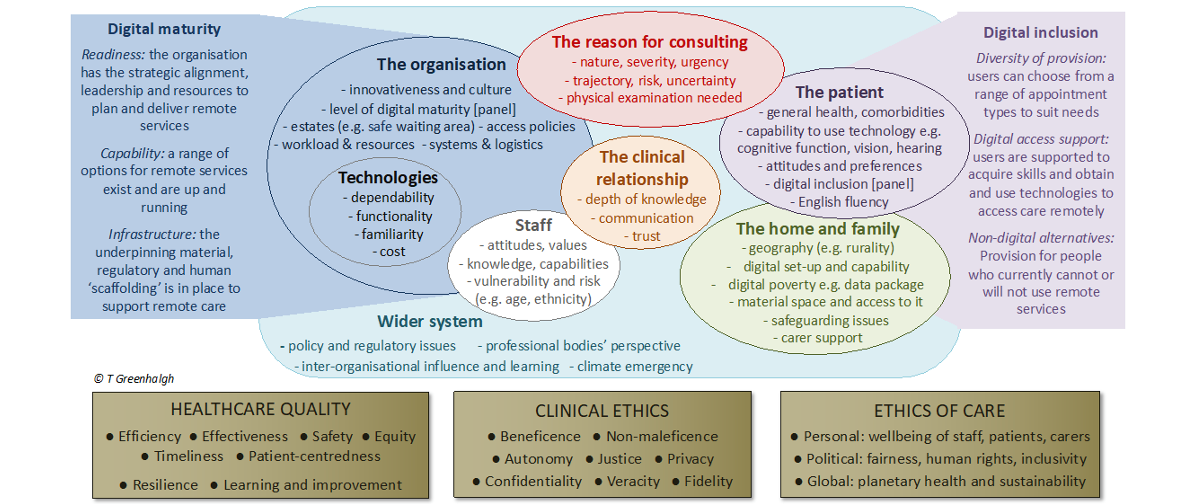
Planning and Evaluating Remote Consultation Services (PERCS) framework (reproduced from Greenhalgh et al [31], with permission from Greenhalgh, T).

We used the Planning and Evaluation of Remote Consultation Services (PERCS) framework [31], which includes a specific focus on a wider system and consideration of climate emergency (Figure 1), to guide synthesis. Sensitized by PERCS, we worked inductively to surface the opportunities and challenges for environmental sustainability presented by virtual consulting services. We then worked deductively to examine if and how any of the 8 domains were identified as relevant to developing sustainable virtual consulting services. One author (MPS) piloted and refined this process on one paper, and we discussed the findings as a team. We then extended the process across the rest of our data set. Using content analysis, we compiled a descriptive overview of the opportunities and challenges identified in the papers and then synthesized and interpreted our findings using the PERCS framework [31].

### Quality Assessment

To our knowledge, there is no checklist currently available to guide the assessment of carbon footprinting studies. Our understanding of the quality of the articles was informed by using the Mixed Methods Appraisal Tool for mixed methods studies [40], which is designed for the appraisal stage of reviews that include qualitative, quantitative, and mixed methods studies. Given that Mixed Methods Appraisal Tool (like other currently available critical appraisal tools) does not apply specifically to articles reporting carbon footprinting, we elected not to exclude articles on the basis of quality appraisal of other study methods but took this into account in the interpretation of their findings.

## Results

### Study Selection

**Figure 2:**
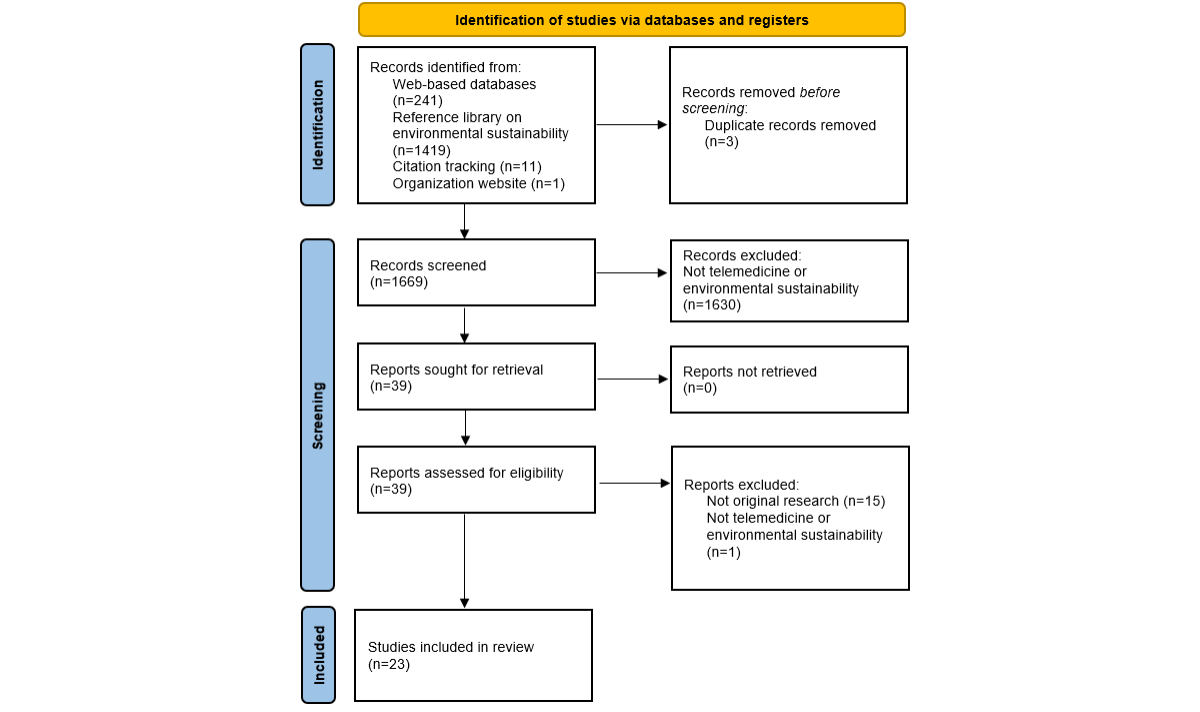
Study flow diagram representing search and screening process.

The database search identified 241 results (Figure 2), with 18 titles relevant to virtual consulting and environmental sustainability in health care. Other methods identified 1431 results with 24 relevant titles. As 3 were duplicates, these were excluded. We reviewed the remaining 39 full-text papers, excluding a further 16 based on relevance or format (Figure 2).

This process provided a total of 23 papers for the review (refer to Table 1 for an overview).

**Table 1:** Overview of studies.

Study	Year in which the study was conducted	Study setting and specialty	Service model and patients	Technology used	Research design and data collected	Key findings
Beswick et al [41]	August 2013 to March 2015	VHA^a^, head and neck cancer, United States	Remote access to tertiary otolaryngology for patients with head and neck cancer involving (1) tissue diagnosis and image acquisition at remote site; (2) multidisciplinary review; and (3) preoperative teleconference with the patient, nurse, and speech pathologist at the patient’s site and the surgeon at the other end, as well as postoperative telemedicine visits as necessary	Teleconferencing	Feasibility study looking at the use of teleconferencing to remotely evaluate 21 (mean age 64 years, all male) patients with head and neck cancer, with clinical, pathological, and operative data collected from electronic patient records, plus costs (of travel and procures) from the VHA finance systems. A total of 26 additional patients were included to allow for comparison with those receiving in-person evaluation.	Telemedicine may expedite treatment planning and operative management. For the 21 telemedicine patients, >US $19,000 was saved between patients and the VHA, and 600 hours were spared on travel to the tertiary care center. An estimated total of 14.5 metric tons of CO_2_^b^ was saved, with average patient savings of 28 hours of traveling, >1600 miles, and US $900 in travel-related costs.
Blenkinsop et al [42]	March to September 2020	Specialist center for neurology and neurosurgery, United Kingdom	Use of telemedicine for patients with complex epilepsy delivered via virtual (replacing in person) clinics during the COVID-19 pandemic	Teleconferencing software (unspecified) and telephone	Calculation of carbon emissions saved by conversion to telemedicine, involving a review of 1567 consultations, representing 1277 adult patients needing specialist expertise. It also accounted for the calculation of emissions directly from the service plus review of clinical records.	Emissions of 35,000-45,000 kg of CO_2_e^c^ were avoided over the study period (6.5 months), largely because of patient travel. This figure accounts for teleclinics emissions (approximately 0.5% of the carbon costs associated with in-person clinics). Benefits of “enforced” telemedicine were noted during the COVID-19 pandemic, with only 1 adverse outcome recorded (inability to review seizure and drug charts via telephone).
Cockrell et al [43]	March 2020 to March 2021	Single quaternary pediatric surgical care facility, United States	Use of telehealth in pediatric surgical and preanesthesia clinics. Telehealth was provided with either a telephone call or telemedicine visit (conducted with videoconferencing software built into the hospital system’s electronic health record)	Telephone or videoconferencing	Retrospective cohort study of 60,773 in-person and 10,626 telehealth encounters of pediatric patients seen by a surgical or preanesthesia provider. This study measured patient-miles saved and CO_2_ emissions prevented to quantify the environmental impact of telehealth. In addition, a regression model was used to assess relationships among patient demographics, geography, and telehealth use.	There was an 8755% increase in telehealth use compared to the year prior to the study. Telehealth resulted in 887,006 patient-miles saved and 688,317 fewer pounds of CO_2_ emitted. Further distance from the hospital and a higher area deprivation index were associated with increased telehealth use (incidence rate ratios 1.0006 and 1.0077, respectively).
Connor et al [44]	2007	University hospital renal service, United Kingdom	Use of virtual consulting to provide routine follow-up to renal transplant recipients, providing quarterly clinic appointments (3 by phone and 1 in person)	Telephone	Prospective study of 30 patients attending 2 consecutive telephone clinics, calculating emissions saved from return journeys (home/hospital) based on postcode and mode of transport. In addition, it calculated staff travel avoided to outlying clinics and carbon savings from reductions in building energy use.	A total of 1180.10 km was saved in patient travel, with a mean CO_2_e reduction of 8.05 kg per patient. Extrapolated to clinic level, this gives an estimated 2818 kg of CO_2_e reduction for 350 consultations pa^d^. Additional reductions through staff travel were estimated at 231.8 kg of CO_2_e pa. Overall carbon saving was estimated at over 3 tons of CO_2_e.
Connor et al [45]	January 2015 to December 2018	Urology service at a single tertiary center, United Kingdom	VC^e^ with electronic guidelines followed by referring clinicians with referral reviewed every day by a urologist or specialist nurse. Telephone consultation was then conducted by a specialist nurse or consultant urologist, leading to discharge investigations plus further VC or in-person clinic or referral for stone intervention.	Telephone	Prospective evaluation of 1008 patients (majority were of working age, 76% male) referred to the virtual colic pathway during the study period. This involved cost-outcomes analysis, carbon footprinting (based on patient travel), a review of logged adverse events, and stone demographics.	In total, 347 patients were discharged from the VC, and after a further VC encounter, 488 patients proceeded to in-person consultations, whereas 173 progressed to surgical intervention. Two adverse events were logged, 4 patients re-presented, 1 patient was incorrectly referred, and 1 presented to another local center. Direct cost of the VC was £29,232 (US $36,333), whereas the opportunity cost of in-person clinic was £174,384 (US $216,745), providing an estimated cost saving of £145,152 (US $180,412). An estimated 15,085 km of patient travel were avoided, equating to a reduction of 0.70-2.93 metric tons of CO_2_.
Croghan et al [46]	Not reported (study takes place over a 3-month period)	Urology service in an urban tertiary referral unit, Ireland	Use of VCs to substitute in-person urology outpatient appointments	Telephone, with videoconferencing platforms available if preferred	VCs were studied over a 3-month period. Based on patient-reported “usual mode of transport” to the hospital, travel distance, time, petrol and parking costs, and carbon emissions avoided by VCs were calculated. The underlying symptom or diagnosis and the “effectiveness” of the VC were also evaluated.	The use of VCs was associated with an overall travel distance saving of 31,038 miles for patients over the study period, with an average round trip journey of 93.8 miles avoided for each rural-dwelling patient and an average financial saving of £25.91 (US $32.20) per rural-dwelling car traveler. An estimated 1257.8 hours of patient time were saved by the avoidance of travel and clinic waiting times. On the basis of car-traveling patients alone, CO_2_ emissions were reduced by 6.07 tons.
Curtis et al [47]	March to April 2020	Emergency orthopedic clinic referrals at a district general hospital, England	Comparison of the impacts of F2F^f^ versus NF2F^g^ orthopedic clinics on patients, the planet, and financial costs	Telephone	Retrospective cohort study with 261 patients identified as having undergone F2F or NF2F orthopedic consultations. Patients were contacted by telephone to establish their experience, mode of transport, and preference for future consultations. Data were also collected to establish the environmental and financial costs to the patient and the trust.	80% of NF2F patients would be happy with virtual consultations in the future. The mean journey distance was 18.6 miles, leading to a reduction in total carbon emissions of 563.9 kg of CO_2_e. The hospital visit carbon cost (heating, lighting, and waste generation) was reduced by 3967 kg of CO_2_e (58%). The financial cost (petrol and parking) was also reduced by an average of £8.96 (US $11.13) per person.
Midlands and Lancashire NHS^h^ CSU^i^ [39]	N/A^j^	Acute hospital-based outpatient services, United Kingdom	Regional, hospital-based outpatient services with varied levels of adoption of web-based consulting	Video consulting	Scoping exercise involving the use of administrative data plus modeling of potential impact of proportions (5%, 62,529 patients; 10%; and 15%) of follow-up appointments held virtually in specialties with limited and established virtual services.	Estimated projected savings with 10% of outpatient follow-ups across specialties include £5.34 (US $6.64) million gross added value, 5200 hours of appointment time, and £5.52 (US $6.86) of (average) patient travel costs per appointment. Estimated projected reduction in emissions range from 177,845 to 533,535 kg of CO_2_ per year (through reduced travel).
Dorrian et al [48]	Not reported (study takes place over 17 months)	ENT^k^ service, Scotland	Use of ENT tele-endoscopy for remote patients, involving local doctors (trained in naso-endoscopy) connected to consultant otolaryngologist via a videoconferencing unit	Videoconferencing unit, connected to laryngoscope	Feasibility study involving head and neck assessment with 42 patients over 17 months, with all patients questioned about their experience immediately after, and follow-up of the first 20 patients (at 2 and 6 months) to confirm patient safety. Emissions saved were then calculated based on patient travel (road and air; cost-minimization analysis).	There were no clinical or technical issues found. In total, 42 journeys were avoided, resulting in an estimated saving of 123 kg of CO_2_e per journey. Variable cost of in-person versus virtual consultation was approximately £383 (US $476) per patient in person versus £77 (US $96) per patient for the tele-endoscopy clinic, with annual fixed cost of £10,502 (US $13,054) for the tele-endoscopy clinic. The threshold at which tele-ENT care became cheaper than travel was a workload of 35 patients pa (actual workload during the study was 29 patients pa).
Dullet et al [49]	July 1996 to December 2013	Cross-specialty outpatients, university hospital, United States	Hospital-based telemedicine program covering outpatient and inpatient interactive video-based consultations, involving >30 clinical specialties to >120 locations across California, with an emphasis on ensuring access for rural and underserved populations	Video consulting	Retrospective analysis of 19,246 outpatient telemedicine consultations (11,281 patients) over 17.5 years. Travel cost savings and environmental impact determined by calculating the difference in mileage costs and emissions between those incurred for in-person appointments and those that would have been incurred had a visit to a client site been needed.	Telemedicine visits saved 5,345,602 miles in total travel distance, equating to total travel time savings of 8.96 years and US $2,882,056 saved directly from travel. The mean per consultation round trip distance savings was 278 miles, with average travel time savings of 245 minutes and US $156 in cost. Total emission savings were 1969 metric tons of CO_2_, 50 metric tons of CO^l^, 3.7 metric tons of NO_x_^m^, and 5.5 metric tons of volatile organic compounds.
Filfilan et al [50]	14 consecutive days in May 2020	Two academic urology departments, France	Use of teleconsultations to replace in-person consultations in a dense city during the COVID-19 pandemic	Live video appointment using Doctolib [51]	Prospective study of all patients (total: 80) who had a remote teleconsultation over 2 weeks during the COVID-19 pandemic. Demographic data were collected to calculate the reduction in CO_2_e emissions from web-based versus in-person appointments. Reduction in travel distance and time were also calculated from these data.	Cars were the usual means of transport. CO_2_e avoided due to the lack of travel was calculated at 1.1 tons. Overall, the total reduction in GHGs^n^ from teleconsultation was 1141 kg of CO_2_e, representing a 99% decrease in emissions. Total savings on transport were €974 (US $1067), and savings on travel time were 112 hours (1.4 hour per patient).
Holmner et al [52]	2005 to 2006 and 2012	Specialist rehabilitation services, Sweden	Hospital-based tele-habilitation program, run by 2 specialist units actively using telemedicine: hand and plastic surgery and speech therapy. Some teleconsultations were in the patients’ homes and others were at a local hub.	Videoconferencing	Life cycle inventory examining the factors contributing to carbon emissions of setting up and running videoconferencing (including, eg, devices and energy used), involving 238 patients who had undergone hand and plastic surgery and 481 patients who had undergone speech therapy.	Telerehabilitation activities of the 2 clinics resulted in a 40 to 70 times decrease in carbon emissions, compared with that of traditional (in-person) rehabilitation. Consultation duration, bandwidth, and use rates also influenced emissions to various extents. Telemedicine became a greener choice (over in-person visits) at a distance of 3.6 km, when the alternative was patient transport by car.
Jiang et al [53]	March to June 2020 and August 2020 to January 2021	VHA, United States	Use of teleoncology visits for veterans during the COVID-19 pandemic, with a particular focus on rural provisions	Audiovisual (computer- or smartphone-based video) or audio- (phone) based medical, surgical, or radiation oncology telehealth services	Patients with experience of teleoncology visits with medical, surgical, or radiation oncology were identified retrospectively. An initial survey was undertaken with 100 patients to determine patient satisfaction with teleoncology services. A follow-up survey was then distributed to 53 of these patients who had had further teleoncology visits. Travel distance, time, cost, and CO_2_ emissions were calculated based on zip codes.	Patients were overall satisfied with teleoncology but felt less satisfied than in-person visits. Audiovisual component improved patient perception of these visits. Follow-up survey demonstrated similar satisfaction. From the 560 teleoncology encounters between March and June 2020, the total travel-related savings were as follows: 86,470 miles, 84,374 minutes, US $49,720, and 35.5 metric tons of CO_2_e.
Lewis et al [54]	October 2006 and October 2007	Regional cancer service, Wales	Telemedicine service to support multidisciplinary teams to improve cancer services	Videoconferencing	Questionnaire completed by users of videoconferencing equipment at 1 site in October 2006 and October 2007 aimed at quantifying reductions in travel time, costs, and emissions.	60 and 90 people reported using the telemedicine service in 2006 and 2007, respectively. Estimated car travel avoided was 18,000 km in 2006 and 20,800 km in 2007. Estimated costs (related to travel) saved was £4400 (US $4819) in 2006 and £5100 (US $5586) in 2007. Estimated emissions avoided was 1696 kg of CO_2_e in 2006 and 2590 kg in 2007.
Masino et al [55]	October 2008 to March 2009	Cross-specialty outpatients, university hospital, Canada	Multisite, hospital-based service providing outpatient teleconsultations to 25 specialties, using dedicated telemedicine hubs	Videoconferencing	Calculation of reductions in travel distance and emissions for 840 telemedicine consultations (covering 615 postcodes and 88 telemedicine locations; 30% of these are surgical critical care assessments) for vehicle and videoconferencing unit energy use, plus cost avoidance.	Estimated total of 757,234 km of travel, 185,159 kg of CO_2_e, and 360,444 g of other air pollutants saved. Energy consumption of videoconferencing units was estimated to be 42 kg of CO_2_e.
Miah et al [56]	July to October 2017	Urology, United Kingdom	Hospital-based virtual urology clinic, running weekly and used for follow-up	Telephone	Prospective study of environmental, clinical, and financial outcomes in 33 VCs, involving 409 patients (55.5% female).	High reported levels of satisfaction, and no reported adverse events with the virtual service. Cost savings were £18,744 (US $23,301) over the 4-month study period (£56,232 [US $69,902] predicted annual savings), with additional income projected via additional in-person capacity (due to shift to virtual service). Estimated travel avoided was 4623 miles; with an estimated 0.35-1.45 metric tons of CO_2_e (depending on mode of transport) avoided over the study period.
Mojdehbakhsh et al [57]	March to April 2020	GynOnc^o^ cancer clinic at University of Wisconsin School of Medicine and Public Health, United States	Quality improvement project, aiming to convert 50% of all outpatient gynecologic oncology encounters during the COVID-19 pandemic to telemedicine within 1 week	Telephone	The Telehealth Satisfaction Survey was administered to 192 telemedicine patients. Descriptive statistics and run charts were used to analyze and depict results. Reduction in CO_2_ emissions was calculated from patients’ home addresses, assuming they travel to the clinic by car.	Patient satisfaction with telemedicine services was high. In addition, 6.25 metric tons of CO_2_ emissions from travel were prevented from being produced during the study period.
Morcillo Serra et al [58]	2020	Nationwide private health care company, Spain	Large hospital network with 29 health care facilities and teaching hospitals, with 3136 doctors from 35 specialties available. Professionals have clinical protocols for the use of digital consultation by specialty and offer the health system 24/7.	Mobile app and website offer various digital health solutions. Video consultations and telephone consultations are also available.	A retrospective study analyzing the environmental impact of the digital health activity of the company’s insurance policyholders and a review of statistical data, working alongside the Carbon Trust.	A net total of 6655 tons of CO_2_ emissions were saved by a reduction in patient travel to surgeries and medical clinics because of the availability of digital appointments and digital access to test results and medical reports. During 2020, a total of 640,122 digital appointments were carried out by the health care company, which avoided 1957 net tons of CO_2_ emissions, whereas patients downloaded 3,064,646 digital medical reports through the company portal, avoiding an additional 4698 net tons of CO_2_ emissions.
Oliveira et al [59]	2004 to 2012	Hospital-based outpatient clinics, Portugal	Real-time outpatient appointments using video to connect patients visiting their GP^p^ to remotely located consultants	Videoconferencing	Survey of a random sample of 100 teleconsultation and 100 F2F outpatients in neurology, dermatology, physical and rehabilitation medicine, and general surgery plus a review of all 20,824 teleconsultations from 2004 to 2011.	Estimated 95% reductions in distance and emissions were associated with travel, equivalent to 455 tons of carbon emissions (22 kg of CO_2_e per patient).
Robinson et al [60]	January 2010 to December 2012 and September 2014 to August 2015	Pediatrics, cerebral palsy, United States	University-based tertiary referral center, offering presurgery evaluations for children with cerebral palsy (and their families) via telemedicine, requiring questionnaire and x-ray to be sent ahead prior to the virtual appointment	Telephone	Calculation of estimated reductions in miles traveled, in travel expense, and in carbon emissions with 279 patients (average age 8 years, 40% female), 161 of whom had a telemedicine preoperative evaluation, plus a further review to determine accuracy and effectiveness from 2014-2015.	Estimated travel reductions via teleconsultation were 106,070 miles (over 3 years) and 658 miles per family, with cost reductions of US $55,326 and US $343,64, respectively, over 3 years (study time). Estimated reductions in emissions were 43,595 kg of CO_2_ over 3 years or 14,532 kg of CO_2_ per year. For each increase of 10 miles from the health center, the odds of a person using phone screening increased by 10%.
Vidal-Alaball et al [61]	January 2018 to June 2019	Primary care services (dermatology, ulcers, eyelids, and audiometrics), Spain	Existing telemedicine services for teledermatology, teleulcers, teleeyelids, and teleaudiometry programs involving electronic transfer of imaging (where relevant) and clinical notes to specialist, with follow-up phone call to patient	Telephone plus imaging	Retrospective study using administrative data to look at reductions in F2F visits and emission of atmospheric pollutants due to 12,322 telemedicine referrals across the 4 relevant services.	A total of 9034 F2F visits were avoided. Estimated reductions included 192,682 km of travel, 3779 hours of travel time, and 11,754 L (€15,664 [US $17,100] in cost) of fuel, resulting in a reduction of 29,384 tons of CO_2_, 36.61 kg of CO, 43.93 kg of N_2_O^q^, and 28.9 kg of SO_2_^r^.
Whetten et al [62]	May 2015 to July 2017	University hospital, neurosurgery department, United States	Use of two-way audiovisual technology plus digital imaging to provide rural patients access to specialist services 24 hours per day, over 365 days per year	Two-way audiovisual technology plus digital imaging	Calculation of avoided emissions for 2020 teleconsultations across 12 rural hospital sites in 1 region plus estimation of scale-up of the service if similar programs operated nationally. Emissions associated with telemedicine use were measured as electricity demand.	A 70% reduction in patient transfers (based on air travel) estimated a total reduction in emissions of 618,722 kg of CO_2_e, with 32 kg of CO_2_e emitted from telemedicine equipment. National expansion of the program was estimated to avoid 213,279 metric tons of CO_2_e.
Wootton et al [63]	2007-2008	Head and neck cancer, minor injuries service, Scotland	Use of video link to (1) connect a nurse or patient at 14 minor injuries units with a hospital-based specialist and (2) connect island-dwelling patients with suspected head and neck cancer with mainland specialists	Videoconferencing	Use of administrative data to calculate reduced emissions through avoided (air and road) travel for 2061 minor injuries (2007) and 42 head and neck cancer (2007-2008) teleconsultations, plus estimations of other potential benefits.	Estimated reductions were 260,000 km of travel and 55 tons of CO_2_ per year for minor injuries services and 3.7 tons of CO_2_ per year for head and neck cancer services.

^a^VHA: Veterans Health Administration.

^b^CO_2_: carbon dioxide.

^c^CO_2_e: carbon dioxide equivalent.

^d^pa: per annum.

^e^VC: virtual clinic.

^f^F2F: face-to-face.

^g^NF2F: non–face-to-face.

^h^NHS: National Health Service.

^i^CSU: Commissioning Support Unit.

^j^N/A: not applicable.

^k^ENT: ear, nose, and throat.

^l^CO: carbon monoxide.

^m^NO_x_: nitrogen oxides.

^n^GHG: greenhouse gas.

^o^GynOnc: gynecologic oncology.

^p^GP: general practitioner.

^q^N_2_O: nitrous oxide.

^r^SO_2_: sulfur dioxide.

### Study Characteristics

The studies reviewed were based on data collected from 1996 to 2021. Eight studies were conducted in the United Kingdom, 7 in the United States, and 2 in Spain, with the remainder in France, Ireland, Sweden, Canada, and Portugal. One study examined the impact of video consulting at a national level across different health care specialties [58]. Two other studies attempted to extrapolate from the analysis of the reduction in carbon emissions in 1 telemedicine service to the national level [39,62]. When combined, these studies provided a broader understanding of the potential reach of virtual consulting in informing the agenda for sustainable health care, including the potential for reduced pollution.

All 23 papers described virtual consulting in terms of telemedicine, teleconsultations, or virtual clinics (Table 1), capturing the use of telephone or videoconferencing equipment (eg, Attend Anywhere) to connect patients with clinicians or primary care clinics with higher levels of care. In 1 instance, videoconferencing equipment was used alongside a companion device (laryngoscope [48]). Two studies used videoconferencing software combined with digital imaging [61,62]. In 1 instance, both telephone and videoconferencing were used to deliver virtual services [42], and in 4 other studies, patients were given the option of either telephone or audiovisual/video consulting [43,46,53,58].

All studies focused on the environmental sustainability potential of telemedicine through the carbon savings achieved by a reduction in travel to face-to-face appointments. The key terms used reflected this, with issues of environmentally sustainable health care framed in terms of reduction in “carbon emissions.” A handful of papers included additional data on other “greenhouse gas emissions” (eg, [61]), whereas approximately half of the studies (11/23, 48%) chose to display these data as a CO_2_e figure (eg, [42,44,47,48,50,53-56,59,62]). None of the papers included in the review provided data on the current footprint of providing their service but instead focused on the impact of virtual consulting on carbon emissions or comparison of emissions via in-person and virtual consulting.

The clinical use of video consulting varied. Some studies focused on the use of video consulting across a range of acute and community clinical settings [39,49,53,55,58,59,61]. The remaining papers focused on local or regional services tied to specific specialties, including urology [45,46,50,56]; neurology and neurosurgery [42,62]; orthopedics [47]; cancer [41,54,57,63]; ear, nose, and throat [48], renal [44], and specialist rehabilitation services [52]; and pediatrics [43,60]. No study focused solely on virtual consulting services in primary or community settings. Most papers (22/23, 96%) looked at telemedicine use within specific medical specialties (eg, neurology and oncology) or at cross-specialty referrals. Across all papers, 12 (52%) looked at telemedicine use in patients who already had a diagnosis, whereas the remainder (n=11, 48%) looked at telemedicine use for patient diagnosis (eg, assessment and imaging done remotely and then communicated to specialists via telemedicine consultations) or first-contact consultations.

### Evidence on Carbon Savings

The language, measurements, and technical processes related to calculating and presenting data on carbon emissions varied across studies. Approximately half of the studies (11/23, 48%) reported estimated carbon emissions in kilograms of CO_2_e, a common unit used to describe all GHG effects as the equivalent global warming potential of carbon dioxide [42,44,47,48,50,53-56,58,62]. Other studies described a simple measure of CO_2_ emissions [39,41,43,45,46,52,57,58,60,63]. Three studies calculated the volumes of other air pollutants or GHGs, such as nitrous oxide and sulfur dioxide [49,55,61]. Papers reported carbon savings using different units, for example, metric tons, tons, kilograms, or pounds, and for different sample sizes of telemedicine consultations and across varying time periods. Only 7 papers reported carbon savings per individual patient consultation [44,47,48,50,59,61,62].

All 23 studies focused on estimating the carbon savings achieved by reducing the amount of road or air travel to in-person appointments (Table 2). Only 1 study included carbon savings associated with an in-person outpatient visit, for example, clinic lighting and heating [47].

Across the 23 studies, the estimated total carbon emissions saved varied greatly because of the focus on anything from a specific service or specialty to regional or national health provision (Table 1). The quantity of carbon savings estimated across studies largely depended on the distance traveled by patients and the mode of transport typically used to get to in-person appointments (eg, cars, buses, trains, or planes). Eight studies conducted surveys or asked patients about the distance they traveled and their preferred mode of travel during consultations [44-47,50,54,56,59]. The remaining studies obtained data to determine the distance traveled from patients’ home addresses listed in patient records, national data, or by determining distances between referral health care facilities. Studies that did not specifically ask patients about their preferred mode of transport typically assumed that car travel would be used and used varied sources to approximate car type, size, fuel type, and driving conditions as the basis of their calculations. For instance, Blenkinsop et al [42] modeled scenarios on cars using petrol only, diesel only, and proportions of petrol and diesel based on the current UK figures for cars sold, excluding 12 consultations that would have involved journeys via air or ferry. Vidal-Alaball et al [61] modeled their data using equal numbers of petrol and diesel cars. Miah et al [56] calculated the difference in savings if people relied on personal car versus public transport (underground train). Three studies also included air travel [41,48,62] in their calculations of transport emissions. Only 1 study included potential carbon savings from reduced staff travel [44].

In studies that used data to calculate commuting distances, specific tools (eg, GPS, Google Maps, and Esri ArcGIS) were used to calculate the approximate distance from the patient’s postcode or between health care referral facilities and assumed that the shortest or quickest route was taken.

The methods used to calculate the carbon footprint varied across studies. Four studies used preexisting web-based carbon footprint calculators [45,46,54,56], the most common being Carbon Footprint Ltd. Other studies manually calculated the carbon savings by multiplying the determined distance traveled by the emission factors for the various modes of travel obtained from emission conversion factor databases, such as the Environmental Protection Agency database in the United States or the Department for Environment, Food and Rural Affairs in the United Kingdom.

**Table 2:** Summary of estimated carbon savings across studies reviewed.

Study	Carbon cost of telemedicine consultation	Total carbon savings of telemedicine throughout the study period	Carbon savings per consultation or patient in the study	Method used to calculate the carbon savings of telemedicine appointments
Beswick et al [41]	Not calculated	14.5 metric tons of CO_2_^a^ (21 patients having 39 telemedicine visits)	Not calculated	EPA^b^ formula (not specified if manually calculated or determined using an EPA web-based calculator); based on road travel in car or light truck
Blenkinsop et al [42]	From 2 kg of CO_2_e^c^ (for telephone calls) to an upper estimate of 167 kg of CO_2_e (for PC setup) for 1152 hours of videoconferencing	35,000-40,000 kg of CO_2_e (1277 patients over 6.5 months)	Not calculated	Manually calculated using emission conversion factors for different types of cars and fuel from the UK Department for Business, Energy, and Industrial Strategy. Distance traveled was calculated using ArcGIS (Esri) and Google Maps.
Cockrell et al [43]	Not calculated	688,317 pounds of CO_2_ (10,626 telehealth encounters)	Not calculated	Miles of travel saved calculated and emissions prevented determined by assuming 25 mile/gallon fuel efficiency and 19.4 pounds of CO_2_ produced per gallon of gasoline (EPA)
Connor et al [44]	Not calculated	3 tons of CO_2_e saved in total: 2818 kg of CO_2_e (350 patient consultations per annum) and 231.8 kg of CO_2_e (staff travel per annum)	8.05 kg of CO_2_e/patient	Distance traveled calculated using Google Maps. Emissions calculated using the DEFRA^d^ conversion factors specific to the mode of transport that patients in the study specified they used.
Connor et al [45]	Not calculated	0.7-2.93 metric tons of CO_2_ (1008 patients between January 2015 and December 2018)	Not calculated	Distance traveled determined using Google Maps. Mode of travel determined from patient (either underground train or car). Carbon footprint calculated using the “Carbon Footprint” calculator.
Croghan et al [46]	Not calculated	6.07 tons of CO_2_ (736 virtual consultations)	Not calculated	Travel distances calculated using AA^e^ Route Planner and Google Maps. Patients’ usual mode of transport obtained during clinical consultations. An average petrol car used as the prototype for car travel. Emissions estimated using the web-based calculator “Carbon Footprint Ltd.”
Curtis et al [47]	Not calculated	6409.9 kg of CO_2_e (saved travel and outpatient emissions, eg, light and heating, for 104 patients who attended virtual consultations)	3.1 kg of CO_2_e/person for travel emissions	Mode of transport and distance from hospital inquired from patients. Emissions calculated using data from a UK government website [64] providing greenhouse gas conversion factors.
Midlands and Lancashire NHS^f^ CSU^g^ [39]	Not calculated	177,845 kg of CO_2_ (if 5% of follow-up appointments across all specialties used teleconsultation); 533,535 kg of CO_2_ (if 15% of follow-up appointments across all specialties used teleconsultation)	Not calculated	Average distance traveled by patients to health facilities in West Midlands was determined, assuming car travel only. The emission conversion factor of 121.10 g of CO_2_/km was used (source not mentioned).
Dorrian et al [48]	Not calculated	Not calculated	123 kg of CO_2_/patient return journey	Distance traveled by road determined using AA Route Planner and between airports using the Vincenty formula. Emissions savings determined according to the methodology of DEFRA.
Dullet et al [49]	Not calculated	1969 metric tons of CO_2_, 50 metric tons of CO^h^, 3.7 metric tons of NO_x_^i^, and 5.5 metric tons of volatile organic compounds (11,281 patients having 19,246 telemedicine visits)	Not calculated	Distance traveled determined from patient’s home address to facility using MapPoint 2013 (Caliper) and MP Mileage 2.5 (Winwaed Software Technology LLC). Average Californian travel speeds were used. Emissions determined from the EPA Average Annual Emissions and Fuel Consumption for Gasoline-Fueled Passenger Cars and Light Trucks report.
Filfilan et al [50]	1.1 kg of CO_2_e per 20-minute consultation using 2 computers	1141 kg of CO_2_e (80 patients over 1 month)	0.5 kg CO_2_e/patient based on energy use of 1 computer for the consultant (not including travel)	Distance traveled determined using Google Maps. Patient’s mode of transport inquired from patient. Cars were assumed to be average car using diesel fuel. Emissions determined using the Greenhouse gas reporting conversion factors on a UK government website [64]. For public transport emissions, national French railway company carbon emission value was used.
Holmner et al [52]	Between 1.86 and 8.43 kg of CO_2_e for a 1-hour telemedicine appointment	40,258-79,909 kg of CO_2_e (481 speech therapy appointments); between 21,400 and 42,472 kg of CO_2_e (238 hand and plastic surgery telemedicine appointments)	Not calculated	Travel distances estimated as the distance from the town closest to the patient’s place of residence to the hospital. Car was used as the transport option. Life cycle carbon costs of traveling addressing tail-pipe emissions; energy consumption; and carbon emissions generated during manufacturing, fuels, and road infrastructure based on research by Lenzen [65] (0.86 kg of CO_2_e/km) and Leduc et al [66] (0.25-0.27 kg of CO_2_e/km).
Jiang et al [53]	Not calculated	35.5 metric tons of CO_2_ (560 teleoncology encounters conducted between March 2020 and June 2020)	Not calculated	Distance traveled determined using Google Maps. EPA conversion factor of 411 g of CO_2_/mile was used.
Lewis et al [54]	Not calculated	1696 kg of CO_2_ (60 patients attending 21 telemedicine appointments in October 2006); 2590 kg of CO_2_ (90 patients attending 30 telemedicine appointments in October 2007)	Not calculated	Distance traveled and mode of transport inquired from patients. Emissions calculated using the UK government calculator.
Masino et al [55]	0.02 kg of CO_2_e per hour for a single device	185,159 kg of CO_2_e; 360,444 g of air pollutants (PM^j^, SO_x_^k^, and NO_x_) avoided (840 telemedicine consultations over 6 months)	Not calculated	Distance traveled determined using Google Maps. Car travel was assumed. Emissions calculated using emission coefficients from Metrolinx.
Miah et al [56]	Not calculated	0.35-1.45 metric tons of CO_2_e (409 patients across 33 virtual clinics over a 4-month period)	Not calculated	Distance determined using Google Maps. Mode of transport inquired from patients. Car used for travel was assumed to be an average 1800cc petrol engine car. Carbon footprint determined using the “Carbon Footprint” calculator.
Mojdehbakhsh et al [57]	Not calculated	6.25 metric tons of CO_2_ (192 patients attending telemedicine visit over a 4-week study period)	Not calculated	Tool used to determine distance traveled was not mentioned. EPA estimate of 4.03×10^−4^ was used to determine emissions.
Morcillo Serra et al [58]	Not calculated	6655 net tons of CO_2_ (640,122 digital appointments and 2,064,646 digitally downloaded patient reports in 2020)	Not calculated	Worked with the Carbon Trust to determine carbon emissions saved. Average distance traveled by patients was determined (tool used not mentioned). Different modes of transport analyzed.
Oliveira et al [59]	Not calculated	455 tons of CO_2_e (for 20,824 teleconsultations)	22 kg of CO_2_e/patient	Distance and modes of transport obtained from patients. Google Maps was used to determine distances. Used average data on types of cars sold in Portugal. Emissions calculated using DEFRA conversion factors, adjusting for average age of cars in Portugal.
Robinson et al [60]	Not calculated	43,595 kg of CO_2_ (161 patients over 3 years)	Not calculated	Distance traveled determined using ArcGIS 10.2 using patients’ home address. Emissions determined using EPA value of 411 g of CO_2_ emissions/mile.
Vidal-Alaball et al [61]	Not calculated	29,384 tons of CO_2_, 36.61 kg of CO, 43.93 kg of N_2_O^l^, and 28.9 kg of SO_2_^m^ (12,322 appointments between January 2018 and June 2019)	3248.3 g of CO_2_/patient journey; 4.05 g of CO/patient journey; 4.86 of g NO/patient journey; and 3.2 g of SO_2_/patient journey	Distance traveled determined using Google Maps. Equal number of diesel and petrol cars was assumed. Emissions were determined by multiplying kilometers by emission factors detailed on the Catalonia Department of Territory website [67].
Whetten et al [62]	0.026 kg of CO_2_e per hour	618,770 kg of CO_2_e (2020 consultations between May 2015 and July 2017); 4307 metric tons of CO_2_e (if statewide); 213,279 metric tons of CO_2_e (if national)	0.306 metric tons of CO_2_e	Distance traveled between rural hospital and transfer site determined using ArcGIS. Bell 429 helicopter model was used. Emissions determined using EPA emission factors, adjusting for higher emissions from air travel.
Wootton et al [63]	Not calculated	55 tons of CO_2_ (2061 teleconsultations in 14 minor injury units in 2007); 3.7 tons of CO_2_ (42 teleconsultations for head and neck cancer in 2007)	Not calculated	Distance from units to main hospital determined (tool used to determine distance was not mentioned). Car travel was assumed. Emissions determined using DEFRA conversion factors.

^a^CO_2_: carbon dioxide.

^b^EPA: Environmental Protection Agency.

^c^CO_2_e: carbon dioxide equivalent.

^d^DEFRA: Department for Environment, Food and Rural Affairs.

^e^AA: Automobile Association.

^f^NHS: National Health Service.

^g^CSU: Commissioning Support Unit.

^h^CO: carbon monoxide.

^i^NO_x_: nitrogen oxides.

^j^PM: particulate matter.

^k^SO_x_: sulfur oxides.

^l^N_2_O: nitrous oxide.

^m^SO_2_: sulfur dioxide.

Two studies extrapolated their findings to a wider area: Midlands and Lancashire NHS Commissioning Support Unit [39] estimated that savings of 533,535 kg of CO_2_ could be achieved if 15% of follow-up consultations in the NHS Midlands (a central United Kingdom region) were made virtual, and Whetten et al [62] estimated that up to 213,279,000 kg of CO_2_e could be saved if neuroemergency consultations were held virtually across the United States before making the decision to transfer a patient to a trauma center.

Five studies [42,50,52,55,62] calculated the carbon footprint associated with telemedicine consultation (Table 2). The estimated savings ranged from 0.002 kg of CO_2_e per hour of consultation (for telephone calls used to deliver virtual care to patients with complex epilepsy during the COVID-19 pandemic) [42] to an upper limit of 8.43 kg of CO_2_e for a 1-hour videoconferencing appointment including energy consumption and embodied emissions of the equipment (eg, design, manufacturing, disposal, and recycling of the equipment) [52].

Four of these studies modeled the carbon footprint of the telemedicine element using a single type of technology [50,52,55,62], with 1 expanding this analysis to make comparisons if patients and doctors used different technologies such as mobile phones, laptops, or personal computers [42]. Although 2 studies referred to additional equipment required for virtual consulting (ie, imaging equipment) [61,62], there was an otherwise limited assessment across papers on the carbon footprint of additional equipment that might be required (eg, headset and software).

### Wider Impact and Development of Virtual Consulting

All reviewed papers concluded, to varying degrees, that virtual consulting significantly reduced carbon emissions. In a handful of papers, authors were able to suggest the point at which virtual consulting offered a greener choice (eg, “at a distance of a few kilometers when the alternative is transport by car” [52]). This led to the overarching conclusion that future health services must be built on sustainable and low-carbon systems and work models that include forms of virtual consulting. In other words, if ambitious targets are to be met [68], then changes to health care practices will be needed at many levels. Although critical and a much-needed ambition in light of the major contribution of health systems to emissions, all papers focused largely, and in most cases (18/23, 78%) exclusively, on patient travel. This clearly represents one of the key areas where reductions in emissions can be achieved (Table 2), particularly in remote and rural communities where distances from providers, emissions, and allied transport costs (eg, via helicopter [62]) are significant. However, patient travel is only one part of the picture, with analysis typically limited in terms of other changes that might be needed, how they might be achieved, and the implications for carbon emissions.

Across the studies, there was limited consideration of wider factors influencing emissions, including wider services and pathways that intersect with and support virtual consulting; the use of (and emissions from) technology enabling health care design and delivery; and wider organizational, infrastructural, and policy-level factors shaping these emissions (all of which involve some form of human or technological activity, with implications for emissions). Consideration of these wider factors was rare and tended to focus on the service or clinic level. For instance, Blenkinsop et al [42] considered emissions at the level of the clinic, focusing on electricity consumption to deliver virtual clinics. They also investigated a sample of patient records and treating clinicians to identify adverse clinical outcomes (n=1). Holmner et al [52] conducted a life cycle inventory to evaluate the carbon reduction potential of telemedicine activities beyond a reduction in travel-related emissions, taking into account end-point devices (eg, computers, monitors, cameras, and local area network components) and video codecs used to compress and decompress digital video signals, and found a 40 to 70 times decrease in carbon emissions compared with in-person visits.

None of the studies (Table 1) assessed the carbon footprint of the entire clinical pathway in which telemedicine consultation was provided as an alternative to in-person visits. This is an important point as virtual consultations do not sit in isolation but are embedded in the health care experienced by patients (and the providers they interact with) and the wider health care system [34]. For instance, although emission reductions were significant from virtual consultations alone, there is potential for further consultations, either virtually or in person, beyond this specific episode of care. There is some evidence that virtual consultations are suitable or appropriate for certain types of conditions or consultations and bring questions about risk and quality for others [69,70], with concerns over potential misses and adverse events. Such instances, although rare [42], potentially bring a need for additional care, at best in the form of a further—likely in-person—consultation and at worst with an admission or more intensive management. Such scenarios were rarely, if ever, considered and potentially negate at least some of the emissions saved from virtual appointments.

Other system-wide considerations [31] were also not included in the papers. For instance, all 23 papers assumed that patients did not need support or care at home (eg, via family) to participate in a virtual consultation (digital inclusion) and that patients who were offered a virtual consultation did not seek other health care or support elsewhere in the system. With the exception of those studies that looked at the carbon savings of virtual consultations between levels of care, for example, clinics and tertiary hospitals [48,55,59,62,63], all the papers assumed that patients remained in their homes to conduct a virtual consultation and did not travel elsewhere (eg, for Wi-Fi access or support from a caregiver). All these scenarios potentially result in increased emissions (although likely small in comparison with reduced emissions through travel). None of these scenarios were considered in the papers included in this review.

## Discussion

### Summary of Main Findings

This review has shown the potential of virtual consulting for helping to address the impact of the climate emergency in health care by contributing to reducing carbon emissions. Most published studies focus primarily (if not, in some cases, exclusively) on reducing patient travel as the main means of achieving this. Our use of the PERCS framework, combined with the systematic search and review of published literature, has enabled us to do the following. First, we have identified the current evidence base on carbon emissions relating to virtual consulting and identified the varied, but consistently sizable, emissions savings enabled by the use of this service model. This ranged from minimal to significant quantities of carbon saved depending on the distance that would have been traveled to the health care facility by patients (and in some cases staff), the method of transport used, the methodology used to calculate the carbon footprint, and the assumptions underpinning it. Second, we have shown that although patient travel is a major consideration, the extent of its contribution varies, for example, between an inner-city primary care clinic (with average travel of <10 km and typically via public transport) and a specialist hospital (serving a regional or wider population with average travel of >100 km by car). Third, we have highlighted that virtual consulting is not a stand-alone interaction but one part of the wider health care system that is rarely considered in papers when thinking about or calculating carbon emissions. Although a focus on patient and staff travel was critical and contributed significantly (albeit variably) to emissions, an exclusive focus on this (episodic sustainability) presented a skewed picture of progress toward system-wide sustainability. Fourth, and related, we have pointed to a range of other factors that are relevant in terms of potential emissions saved and cost in terms of emissions produced via design, provision, and the use of virtual consulting services.

### Comparison With Wider Literature

Our findings resonate with those from an earlier systematic review [35], which clearly demonstrates that virtual consulting has a valuable role to play in achieving lower-carbon health systems. However, this and other literature in the field [71-73] tends to focus solely on carbon emissions allied to travel, with limited analysis of the design and delivery of virtual consulting, limited critical reflection on the assumptions underpinning calculations, and limited focus on the entire clinical pathway. Although it is clear from our own review and that of Purohit et al [35] that reducing travel through the use of virtual consulting holds the potential for significant emissions savings, consideration of patient, carer, organizational, and system-wide factors (Figure 1) shaping the planning and delivery of virtual consulting is needed.

Previous studies have tended to focus on carbon footprinting of virtual consulting in specific services and settings. Some studies have sought to extend findings about reduced emissions from 1 service to the wider health system. Although welcome, care needs to be taken here: not all health care can be provided virtually, and there needs to be reasoned application of it, acknowledging the nature of different clinical presentations as well as increasing the awareness of the environmental impact of the entire clinical pathway in these decisions. Recent work emphasizes this point, highlighting how despite the crisis context and resourcing, video consulting did not pick up in general practice during the COVID-19 pandemic when it was expected to (suggesting that video consulting in the UK NHS at least is not well accepted outside hospital settings) [74]. Our review adds to the existing evidence by shedding light on these wider influences and the need to consider them in future service design and delivery as well as research and evaluation. This requires a multilevel approach to generating evidence that is attentive to multiple considerations spanning policy to practice.

The potential of virtual consulting across health care settings and specialties has been well-documented over the course of the COVID-19 pandemic. Published evidence from ourselves and others [30,31,34,36] indicates that virtual consulting is feasible, broadly acceptable to staff and patients, safe over the short term, and associated with sizable net emission savings. This latter point is a key finding from the review. However, papers say very little about how to develop virtual consulting services nor the policy, regulation, and professional support needed to progress virtual consulting and environmentally sustainable health care. To our knowledge, little has been done at this intersection. There is considerable attention on virtual consulting and sustainable health care and only a small focus on the intersection between them. One recent example is the development of a national-level video consulting program (the Scottish Near Me service) and the drive for more environmentally sustainable health care. Although developing separately in Scotland, these 2 major initiatives have intersected in ways that appear productive, not only allowing for investment in material and technological infrastructure, staff training, and professional and public engagement to support video consultation services [36] but also enabling a parallel focus on emissions reduction.

The carbon savings presented in the studies reviewed seem impressive. However, it is still only a small part of the solution to reducing health care’s carbon footprint. According to the carbon footprint model of the NHS [8], personal travel accounts for 10% of estimated emissions as follows: 5% from patient travel, 4% from staff travel, and 1% from visitor travel. This is in comparison with the medical supply chain, which contributes to 62% of the emissions. Thus, although telemedicine is one way in which the health system can reduce its environmental impact, a focus on reduced travel and increased use of virtual consulting must not be seen as the only solution. Many other innovations and initiatives (see [26,75-80] for examples) need to be developed, adopted, and spread if health systems are to contribute significantly to net zero ambitions. It is likely that digital technology has a major role to play in this. However, caution is required in relation to the “rebound effect,” whereby a more efficient technology results in higher carbon emissions because of the increased use of that technology [80]. This holds potential for increased use of telemedicine and the devices and platforms that support and interconnect with it, to give rise to higher carbon emissions.

Carbon footprinting is a useful tool but remains a relatively new field, with limited mainstream knowledge about the process and evolving quality assessment. A carbon footprint is only a best estimate of the direct and indirect emissions produced by a certain product or scenario and usually includes the 7 GHGs covered in the Kyoto Protocol [81], expressed as CO_2_e. The GHG Protocol outlines 3 scopes that aid in the reporting of all emissions associated with an organization or activity: scope 1, direct emissions from the organization; scope 2, indirect emissions associated with the organization’s electricity use; and scope 3, all other indirect emissions including supply chain emissions [82]. When calculating the emissions from driving a vehicle, one needs to consider the direct emissions from burning the fuel, which are different depending on whether the vehicle runs on petrol, diesel, or electricity; the scope 3 emissions embedded in a vehicle, namely, well-to-tank emissions (the extraction of raw oil, transport to the oil refinery, production of fuel, and transport of fuel); and the GHG emissions associated with manufacturing and distribution. This process requires significant expertise. To estimate emissions, some of the studies reviewed inserted numbers into a web-based carbon calculator: this process typically does not take into account scope 3 emissions, frequently uses different methods to produce carbon footprint values (some of which are poorly explained or reported in the studies using them), are usually only valid in the country in which the calculator was produced, and thus commonly results in some uncertainty over the accuracy and reproducibility of reported carbon savings.

The distances used to calculate the carbon footprint in the studies were generally based on estimates from postcode data, with the assumption that there were normal driving conditions and that patients drove the most direct route to the hospital. This, in addition to other assumptions (eg, type of vehicle or fuel), introduces a level of unavoidable uncertainty. This means that carbon footprints remain an estimate of environmental impact.

Carbon footprint calculations consider the environmental impact from an emissions perspective and do not take into account other toxins released or waste produced from a process. In the case of telemedicine, switching to virtual consultations will require electronic technology, which, when it has completed its life cycle, will likely end up in landfills (eg, the “electronic graveyard” [83]). Other environmental impacts of virtual versus face-to-face appointments were not included in any of the studies reviewed. This remains a gap in the literature.

### Strengths and Limitations

This paper has shone a much-needed light on the potential of virtual consulting as one means by which health systems can reduce carbon emissions and contribute to addressing climate change. Our use of the PERCS framework [31] was helpful in widening the considerations of environmental sustainability beyond carbon emissions through patient travel alone. However, we were limited in the range of papers reviewed, most of which focused on secondary care (or tertiary or quaternary) services and included virtual consulting services provided to outpatients and those with a preexisting diagnosis. This limits the potential transferability of findings (eg, the extent of carbon emissions), particularly with regard to primary care settings. This may have been compounded by our original search strategy, which focused on 3 databases. It is possible that a broader search across other databases would have identified additional papers. We were limited by the quality of the papers, which used varying carbon methodologies, and the lack of critical appraisal tools available to assess the quality of the academic literature on carbon modeling.

### Conclusions

Environmentally sustainable health care aims to facilitate health systems that are able “to anticipate, respond to, cope with, recover from and adapt to climate-related shocks and stresses, while minimising negative impacts on the environment and leveraging opportunities to restore and improve it” (page 26) [10]. The goal is to protect the health and well-being of current and future generations [23,24,52]. Virtual consultations in routine care provision are one means of reducing the environmental impact of health care and can provide significant carbon savings. It is widely accepted that this is due largely (but not exclusively) to the removal of the need for patients to travel to health care facilities. Further work is now urgently needed to fully appreciate the extent of carbon emissions and the potential for reductions (as well as other environmental impacts, eg, electronic waste) across the entire clinical pathway, rather than reductions focused on virtual consultations alone. This research should be conducted across all levels of health care provision, including primary, secondary, and tertiary levels. Those evaluating and funding virtual consulting services need to better appreciate the potential for reductions in emissions and weigh this up in relation to the benefits and potential risks (eg, adverse events and missed diagnoses) associated with providing or scaling up such services. If we are to see a rapid and pronounced change, then we urge both funders and evaluators to include carbon footprint modeling routinely into their design and methods and to fully engage with the transdisciplinary endeavor of reviewing, monitoring, and addressing issues of environmental sustainability.

We encourage all those performing carbon modeling of health care services to apply consistent methodology according to published standards (eg, the GHG Protocol). Policy makers and planners would do well to consider the potential of virtual consultations to help mitigate the effects of climate change, in the context of the wider system changes needed to support the development, adoption, and spread of these services in ways that enable reductions in emissions across clinical pathways while simultaneously enabling access and digital inclusion. This requires investment in climate literacy and skills training. Time is short. We urge funders, evaluators, policy makers, and planners to coordinate; to act with a sense of urgency to address environmental impact in health systems; and, in line with the Paris Agreement, to work toward limiting the mean global temperature rise to well below 2 °C.

## Appendix

Multimedia Appendix 1 Search terms relating to telemedicine and sustainable health care on Ovid MEDLINE, PubMed, and Scopus.

## Abbreviations

CO_2_e: carbon dioxide equivalent
GHG: greenhouse gas
NHS: National Health Service
PERCS: Planning and Evaluation of Remote Consultation Services
PRISMA: Preferred Reporting Item for Systematic Reviews and Meta-Analyses
PRISMA-P: Preferred Reporting Items for Systematic Review and Meta-Analysis Protocols

## Data Availability

Data generated or analyzed during this study are included in this paper and its multimedia appendix file.
