# Supplementary material for: The Role of Virtual Consulting in Developing Environmentally Sustainable Health Care: Systematic Literature Review
Source: J Med Internet Res. 2023 May 3;25:e44823. doi: 10.2196/44823 (PMC10193215; doi:10.2196/44823)
Supplement: Multimedia Appendix 1 [file jmir_v25i1e44823_app1.docx]

## Multimedia Appendix 1

**Search terms relating to telemedicine and sustainable healthcare on Ovid MEDLINE®, PubMed, and Scopus.**

| **Medline (Ovid MEDLINE®)** | 1. telemedicine/ or remote consultation/ 2. (telehealth or tele-health or tele-medicine or mhealth or m-health or mobile health or e-health).ti,ab. 3. (remote consultat* and (telephone? or cellphone? or phone? or iphone? or virtual or video)).ti,ab. 4. (teleconsult* or tele-consult* or teleclinic? or tele-clinic?).ti,ab. 5. ((consult* or appointment*) adj5 (telephone? or cellphone? or phone? or iphone? or virtual or video*)).ti,ab. 6. ((remote or virtual or telephone? or phone? or video*) adj clinic?).ti,ab. 7. 1 or 2 or 3 or 4 or 5 or 6 8. "Conservation of Natural Resources"/ 9. Sustainable Growth/ 10. exp climate change/ or greenhouse effect/ 11. Environmental monitoring/ or environmental indicators/ 12. ((Carbon or CO2) and (emissions? or emit* or pollut* or efficien* or cost? or saving* or footprint*)).ti. 13. ((carbon or co2) adj3 (emissions? or emit* or pollut* or efficien* or cost? or saving* or footprint*)).ti,ab. 14. (global adj (heating or warming)).ti,ab. 15. ((Climate* adj2 change*) or greenhouse effect* or greenhouse gas*).ti,ab. 16. ((Environment* or climat* or planet*) adj2 (footprint? or sustain* or impact*)).ti,ab. 17. ((Sustainab* or green or planetary) adj2 health*).ti,ab. 18. 8 or 9 or 10 or 11 or 12 or 13 or 14 or 15 or 16 or 17 19. 7 and 18 20. limit 19 to english language |
| --- | --- |
| **PubMed** | (("telemedicine" [Title/Abstract] OR "telehealth"[Title/Abstract] OR "tele-health"[Title/Abstract] OR "tele-medicine"[Title/Abstract] OR "m-health"[Title/Abstract] OR "mobile health "[Title/Abstract] OR "e-health"[Title/Abstract]) AND ("greenhouse gas emission*"[Title/Abstract] OR "ghg"[Title/Abstract] OR "carbon footprint"[Title/Abstract] OR "environmental impact"[Title/Abstract] OR "life cycle inventory"[Title/Abstract] OR "life cycle analysis"[Title/Abstract] OR "cradle to grave"[Title/Abstract] OR "environment"[Title/Abstract] OR "green"[Title/Abstract] OR "sustainability"[Title/Abstract]) AND ("hospital*"[Title/Abstract] OR "healthcare*"[Title/Abstract] OR "health care"[Title/Abstract] OR "health service*"[Title/Abstract] OR "medicine"[Title/Abstract] OR "clinic*"[Title/Abstract] OR "patient*"[Title/Abstract] OR "health system*"[Title/Abstract]) AND ("reduc*"[Title/Abstract] OR "recommend*"[Title/Abstract] OR "solution*"[Title/Abstract] OR "green behaviour"[Title/Abstract] OR "net zero"[Title/Abstract] OR "initiative*"[Title/Abstract] OR "guideline*"[Title/Abstract] "guideline*")) |
| **Scopus** | TITLE-ABS-KEY ( ( ( ( "greenhouse gas emission*" OR "ghg" OR "carbon footprint" OR "environmental impact" OR "life cycle inventory" OR "life cycle analysis" OR "cradle to grave" OR "environment" OR "green" OR "sustainability" ) AND ( "hospital*" OR "healthcare*" OR "health care" OR "health service*" OR "medicine" OR "clinic*" OR "patient*" OR "health system*" ) AND ( " telemedicine" OR "telehealth " OR "tele-health" OR "tele-medicine" ) AND ( "reduc*" OR "recommend*" OR "solution*" OR "green behaviour" OR "net zero" OR "initiative*" OR "guideline*" ) ) ) ) AND ( LIMIT-TO ( PUBYEAR , 2022 ) ) AND ( LIMIT-TO ( LANGUAGE , "English" ) ) |
